# Iliopsoas Abscess Possibly due to* Klebsiella pneumoniae* Infection after Chemoradiotherapy for Hypopharyngeal Cancer

**DOI:** 10.1155/2016/1343106

**Published:** 2016-02-16

**Authors:** Yukiyoshi Hyo, Tomoya Fujisaki, Rui Hyo, Hiroki Tanaka, Tamotsu Harada

**Affiliations:** ^1^Department of Otolaryngology, Kawasaki Medical School, 577 Matsushima, Kurashiki, Okayama 701-0192, Japan; ^2^Department of Hematology, Kawasaki Medical School, 577 Matsushima, Kurashiki, Okayama 701-0192, Japan

## Abstract

Iliopsoas abscess was once an uncommon condition but now occurs somewhat more frequently due to the increasing number of immunocompromised patients, such as those with diabetes. We encountered a case of iliopsoas abscess following chemoradiotherapy for hypopharyngeal cancer. A 60-year-old man was admitted for a sore throat and left neck swelling. Hypopharyngeal cancer was diagnosed, but the patient refused surgery. After two rounds of chemotherapy, febrile neutropenia developed and chest computed tomography (CT) revealed an iliopsoas abscess. The platelet count was low but recovered after administration of antibiotics and could not be explained by puncture of the abscess. CT-guided drainage eventually improved his symptoms. Even for disorders of the head and neck region, iliopsoas abscess should be suspected in immunocompromised patients who develop a fever. CT and magnetic resonance imaging should be performed at an early stage as it is important to determine whether surgical drainage is indicated.

## 1. Introduction

Iliopsoas abscess was previously uncommon condition that usually originates from thoracolumbar spinal infection, and its incidence has decreased with advances in chemotherapy. However, the condition has shown a recent resurgence due to the increased number of diabetic, elderly, and cancer patients. As a result, this condition should be carefully considered during routine examinations [1]. There are two types of iliopsoas abscess, primary and secondary, and patients that have been immunocompromised over the long term, such as patients on dialysis or diabetic patients, are commonly susceptible to this condition [2]. Although iliopsoas abscess may also be encountered in head and neck surgery and during treatment, its etiology is not well understood. Here, we report a difficult-to-treat patient whose period of myelosuppression after chemotherapy for hypopharyngeal cancer was complicated by a primary iliopsoas abscess. In addition, we briefly discuss the literature.

## 2. Case Presentation

**Figure 1:**
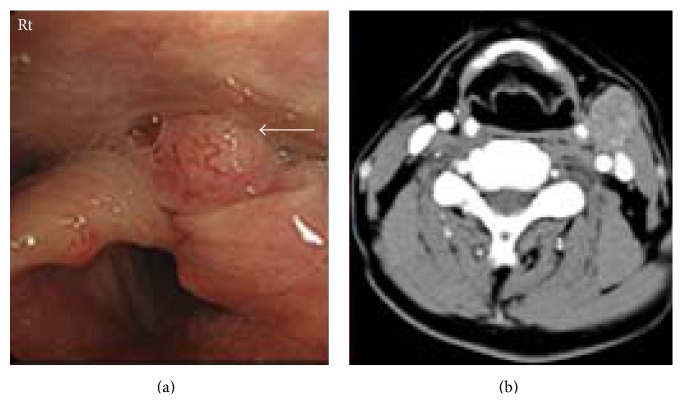
Contrast computed tomography of the neck showing a mass lesion in the left piriform recess, with metastasis to a lymph node on the left.

A 60-year-old man was admitted for sore throat and left neck swelling. A mass lesion was observed in the left piriform recess during endoscopic examination, and the patient was diagnosed as having squamous cell carcinoma. A comprehensive examination led to a diagnosis of hypopharyngeal cancer in the piriform recess (T3N2aM0, Stage IVa; Figure 1). Laboratory analysis performed on admission revealed no abnormalities: hemoglobin, 13.5 g/dL; platelets, 23.3 × 10^4^/*μ*L; aspartate transaminase, 14 U/L; alanine transaminase, 20 U/L; creatinine, 0.90 mg/dL; and blood urea nitrogen, 19 mg/dL. The patient refused surgery and was admitted on August 1 for chemoradiotherapy. Radiotherapy was started the day after admission, and chemotherapy (docetaxel, 60 mg/m^2^; cisplatin, 80 mg/m^2^; and fluorouracil, 500 mg/m^2^) was started 1 week later.

**Figure 2:**
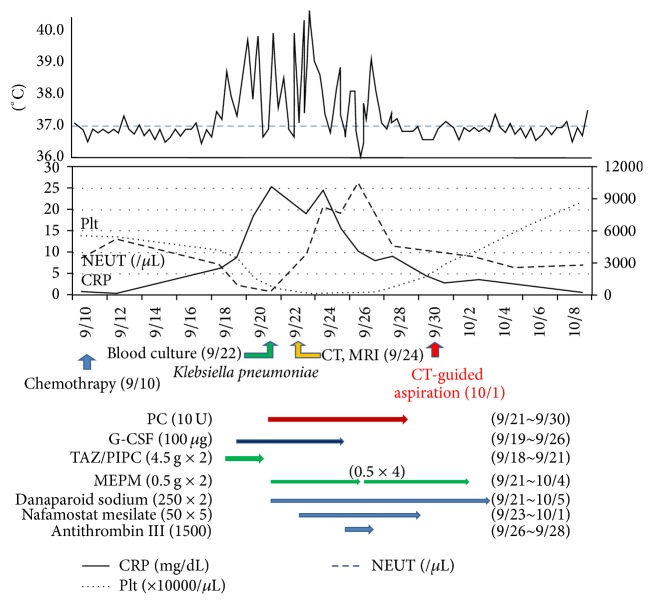
Clinical course of the patient. MEPM: meropenem; PC: platelet concentrate transfusion; G-CSF: granulocyte colony-stimulating factor; TAZ/PIPC: tazobactam/piperacillin.

**Figure 3:**
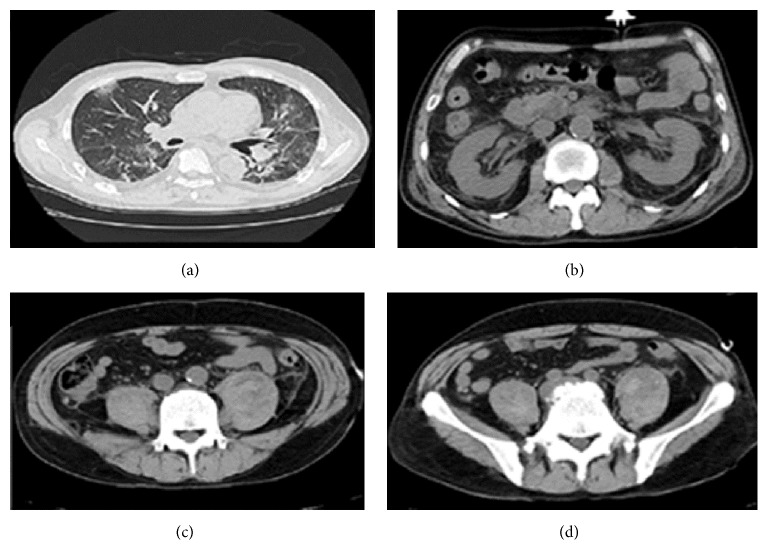
Chest and abdominal computed tomography findings. (a) Mild pneumonia and alveolar hemorrhage. (b) Edema in the retroperitoneum surrounding the kidney. (c) and (d) Bilateral enlargement of the iliopsoas muscles and areas of nonuniform swelling.

**Figure 4:**
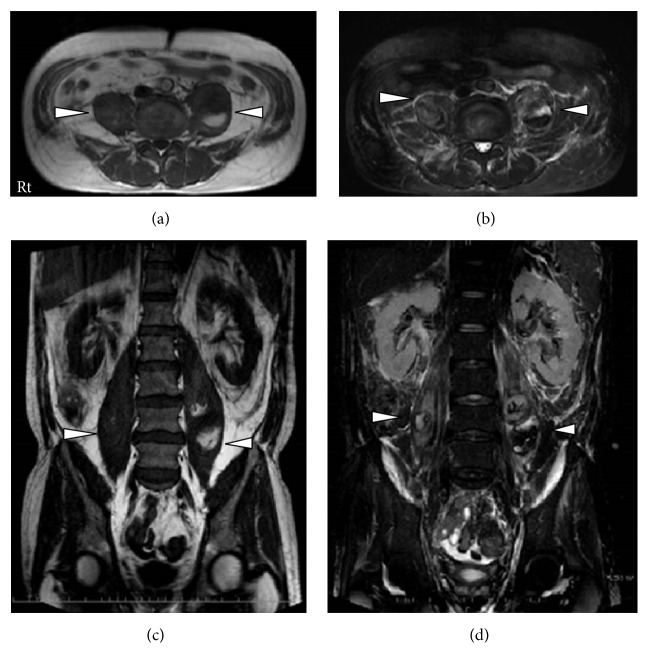
Abdominal magnetic resonance imaging. The bilateral iliopsoas abscesses are indicated by arrow heads. (a) T1-weighted axial image. (b) T2-weighted axial image. (c) T1-weighted coronal image. (d) T2-weighted coronal image.

Myelosuppression was induced after 1 week of chemotherapy, and an intravenous infusion of tazobactam/piperacillin (TAZ/PIPC) 4.5 g was administered 3 times daily. The patient developed transient drug-induced renal dysfunction that resolved spontaneously. On September 10, the patient underwent radiotherapy at a dose of 40 Gy, and a complete response was achieved with a 60% reduction in the size of the cervical lymph nodes. The second round of chemotherapy (switched to docetaxel, 40 mg/m^2^; fluorouracil, 600 mg/m^2^; and carboplatin, 5 mg/m^2^/min) was started on the same day. On the second day after completing chemotherapy, the patient developed a fever and exhibited a decreased white blood cell count. He was treated with granulocyte-colony stimulating factor (100 *μ*g) and restarted on TAZ/PIPC 4.5 g, 3 times daily. However, the fever did not improve. On day 4 after completing chemotherapy, his neutrophil count was 338/*μ*L and his platelet count had decreased to 1.9 × 10^4^/*μ*L. The patient's clinical course is shown in Figure 2. He was switched to meropenem (MEPM; 0.5 g, 2 times daily), and treatment for disseminated intravascular coagulation (DIC) was initiated. Six days later, his neutrophil count had improved to 3380/*μ*L, but his platelet count remained low, at 0.4 × 10^4^/*μ*L. Platelet transfusion was performed the next day. Despite an improved neutrophil count, gastrointestinal hemorrhage was suspected as the cause of the unimproved platelet count. On physical examination, he did not appear to be in poor physical health except for the spiking fever. No signs of infection were seen on urinalysis. The patient therefore underwent gastrointestinal computed tomography (CT) on September 24. Imaging findings showed intraalveolar hemorrhage but no gastrointestinal hemorrhage (Figure 3). However, imaging findings suggested massive bilateral iliopsoas abscesses, which were confirmed on plain magnetic resonance imaging (MRI), and iliopsoas abscess was diagnosed as a complication (Figure 4). Needle aspiration could not be performed because the platelet count was below 1.0 × 10^4^/*μ*L, so the antibiotics and anti-DIC therapy were continued. The platelet count increased to 4.3 × 10^4^/*μ*L on September 30, and the patient underwent needle aspiration of the iliopsoas abscesses under CT guidance on October 1. Thereafter, MEPM was continued and the symptoms improved. The patient was discharged on October 15. He is being followed up for hypopharyngeal cancer, and no local recurrence or recurrence in the cervical lymph nodes has been observed, with a favorable course to date.* Klebsiella pneumoniae* was detected in the blood culture performed on September 22, but not from the iliopsoas abscess puncture performed on October 1. A sensitivity analysis revealed resistance to ampicillin but sensitivity to other antibiotics. Recurrence was checked on CT before the patient was discharged and showed that the iliopsoas abscess had disappeared.

## 3. Discussion

Until recently, iliopsoas abscess was an uncommon condition. The classic triad of pain, fever, and limp that has been described is rarely seen [3]. Iliopsoas abscess is commonly diagnosed with modern imaging techniques, such as ultrasonography, CT, and MRI. These days, there appears to be a relative decrease in the occurrence of this condition due to advances in antibiotics, better general sanitation, and improved nutrition. On the other hand, the number of immunocompromised patients has increased, triggering a recent increase in the disease. The incidence of iliopsoas abscess in the United Kingdom is 0.4 per 100,000 [3], and 118 cases occurred in South Korea over 10 years [4], 61 cases occurred in the United States over 7 years [5], and 82 cases have been reported in Japan over 4 years [6]. Previously, the cause of this condition was commonly tuberculosis. Although tuberculosis is still observed in developing countries, the infection in recent years is more commonly due to methicillin-resistant* Staphylococcus aureus *(MRSA) or* Enterobacter* spp.

The iliopsoas muscle has direct contact with the vertebrae and other organs such as the appendix, colon, small intestine, kidney, ureter, and pancreas. When inflammation spreads directly from these surrounding organs to the iliopsoas muscle and an abscess forms, this is known as a secondary iliopsoas abscess. When the site of infection is more distant, it is known as a primary iliopsoas abscess. Causes of secondary iliopsoas abscess include gastrointestinal diseases such as Crohn's disease or appendicitis, or diseases of other organs, such as the kidney or vertebrae, or even emergent inflammatory diseases of the retroperitoneum and intrapelvic organs that surround the iliopsoas muscle. In all cases, states of immunodeficiency, such as those resulting from malnutrition, diabetes mellitus, severe infection, or administration of steroids, are considered important to the etiology of infection. Santaella et al. [7] considered hematogenous or lymphogenous spread due to latent infection in states of immunodeficiency, which leads to abscess formation. Secondary abscesses are usually more common, with Tabrizian et al. [5] reporting primary abscesses in 7 of 61 cases in a study published in 2009 and Lai et al. [8] reporting primary abscesses in 199 of 522 cases in a literature review published in 2011. It is evident that primary abscesses are less common than secondary abscesses.

Approximately 30% of patients present with the classic triad of symptoms mentioned above, with a spiking fever and low back pain. Considering an iliopsoas abscess in the differential diagnosis is important for early detection, and CT examination is also believed to be important.

The causative organism in primary iliopsoas abscess is usually a Gram-positive* Staphylococcus* species. According to a report by Kim et al. [4], 34 of 105 cases were caused by infection with* Staphylococcus aureus*, and, of them, 12 were caused by MRSA. However, as other studies have detected Gram-negative bacteria, a broad-spectrum antibiotic treatment effective against both Gram-positive and Gram-negative bacteria should be used when considering empirical treatment, even in patients with primary iliopsoas abscess.

Treatment may also involve a conservative approach with antibiotic agents or may require invasive procedures. Invasive interventions include percutaneous drainage and laparotomy, depending on the size of the abscess.If the abscess is >3 cm, the limits of conservative therapy are reached, and surgical drainage should instead be considered [9]. When the present patient was started on antibiotic treatment, the abscess was massive (>3 cm), and although there were no indications for conservative therapy, drainage could not be performed immediately because of the decreased platelet count as a result of DIC, which was simultaneously discovered. For this reason, the patient was treated conservatively using antibiotic agents, the DIC improved, and bilateral percutaneous drainage was performed under CT guidance.

The only report of iliopsoas abscess complicating conditions of the head and neck was by Sunose et al. [10]. They reported an iliopsoas abscess caused by MRSA after surgery for laryngeal cancer and stated that it is possible that this condition may be a poorly understood disease in the field of otorhinolaryngology. Here, we treated a patient who refused surgery for advanced hypopharyngeal cancer and underwent chemoradiotherapy. Thereafter, febrile neutropenia occurred as an adverse drug reaction to the anticancer drugs, and* K. pneumoniae* was detected in the blood culture. Based on this finding, this organism was considered the likely cause of abscess formation in the well-perfused iliopsoas muscle. In addition, we regarded the abscess to be primary because there were no gastrointestinal symptoms or signs of pyogenic spondylitis. In this patient, we continued platelet transfusion and anti-DIC treatment and used the broad-spectrum antibiotic agent MEPN. The patient recovered to the extent that we could perform CT-guided percutaneous drainage. Although we were unable to identify the causative organism, we presumed it to be* K. pneumoniae* based on the blood culture results.

In recent years, the number of patients with underlying diseases, such as diabetes mellitus or renal failure, has increased, and such patients can also be expected to be common among patients with tumors of the head and neck. In addition, chemoradiotherapy is increasingly performed to maintain organ function. Our patient had febrile neutropenia due to chemotherapy, a condition that should be considered in patients who develop DIC, have a decreased platelet count, and show little improvement. Furthermore, this condition should be suspected in an immunocompromised patient with a spiking fever, and CT and MRI should be performed early to confirm whether there are indications for surgical drainage. Moreover, proactive imaging of the thoracic region is recommended.

## 4. Conclusion

Here, we reported a case of hypopharyngeal cancer, subsequently complicated by an iliopsoas abscess, in a patient who developed DIC as a result of chemoradiotherapy. Even in the treatment of otorhinolaryngological conditions, iliopsoas abscess should be suspected when fever occurs in an immunocompromised patient, and CT and MRI investigations should be performed early to confirm whether there are indications for surgical drainage.
